# Seat belt use among rear passengers: validity of self-reported versus observational measures

**DOI:** 10.1186/1471-2458-8-233

**Published:** 2008-07-09

**Authors:** Francesco Zambon, Ugo Fedeli, Maria Marchesan, Elena Schievano, Antonio Ferro, Paolo Spolaore

**Affiliations:** 1 Regional Center for Epidemiology, Veneto Region, Via Ospedale 18, 31033 Castelfranco Veneto, Italy; 2 Regional Department for Prevention, Public Health Section, Rio Novo 3493, 30123 Venezia, Italy

## Abstract

**Background:**

The effects of seat belt laws and public education campaigns on seat belt use are assessed on the basis of observational or self-reported data on seat belt use.

Previous studies focusing on front seat occupants have shown that self-reports indicate a greater seat belt usage than observational findings.

Whether this over-reporting in self reports applies to rear seat belt usage, and to what extent, have yet to be investigated.

We aimed to evaluate the over-reporting factor for rear seat passengers and whether this varies by gender and under different compulsory seat belt use conditions.

**Methods:**

The study was conducted in the Veneto Region, an area in the North-East of Italy with a population of 4.7 million.

The prevalence of seat belt use among rear seat passengers was determined by means of a cross-sectional self-report survey and an observational study.

Both investigations were performed in two time periods: in 2003, when rear seat belt use was not enforced by primary legislation, and in 2005, after rear seat belt use had become compulsory (June 2003).

Overall, 8138 observations and 7902 interviews were recorded.

Gender differences in the prevalence of rear seat belt use were examined using the chi-square test. The over-reporting factor, defined as the ratio of the self-reported to the observed prevalence of rear seat belt use, was calculated by gender before and after the rear seat belt legislation came into effect.

**Results:**

Among rear seat passengers, self-reported rates were always higher than the observational findings, with an overall over-reporting factor of 1.4.

We registered no statistically significant changes over time in the over-reporting factor, nor any major differences between genders.

**Conclusion:**

Self-reported seat belt usage by rear passengers represents an efficient alternative to observational studies for tracking changes in actual behavior, although the reported figures need to be adjusted using an appropriate over-reporting factor in order to gain an idea of genuine seat belt use.

## Background

The effectiveness of seat belt usage in reducing the severity of the sequelae of motor vehicle occupant injuries and fatalities in road accidents is widely known. This also applies to rear seats, where using a seat belt is estimated to reduce the risk of death by 18% to 75% [1-3]. Furthermore, wearing a seat belt in the rear seats is considered effective not only to protect the rear seat passengers, but also to reduce the injuries and fatal consequences to front seat occupants [4,5]. It has been estimated that the risk of a front seat occupant being killed in a frontal impact increases by about three-quarters if there is an unrestrained passenger in the seat behind them [6,7].

With a view to promoting road safety, many countries have made it compulsory to wear seat belts even in the back seats. In the European Union, 21 of 25 countries now require safety belt use in the rear seats [8], but the proportion of rear seat passengers who use seat belts remains lower than for drivers or front seat passengers, and varies considerably among different countries, ranging from 15% to 90% [9,10]. Among low- and middle-income countries, the reported prevalence of seat belt use among rear seat passengers is 1% or even lower, suggesting a need for urgent action [11-13].

To assess the effect of seat belt legislation appropriately and judge the effectiveness of health policies focusing on promoting seat belt use, it is important to properly measure their actual usage, which is estimated mainly from direct observations and self-report surveys. Both methods have their limitations. On the one hand, observational surveys are expensive, they are based on a one-off assessment, and they cannot properly determine seat belt usage in vehicles with tinted windows, to mention some of their weaknesses [14]. On the other hand, self-reported measures have proved unreliable because respondents over-report their use of the seat belt, especially where seatbelt use is mandatory [15,16].

The validity of self-reported seat belt usage estimates has been assessed by comparing them with observational findings, which are usually referred to as the "gold standard" [17,18]. An "individual level" and a "community level" approach have both been used for this comparison: in the former, the consistency between observed and self-reported seat belt use was examined by interviewing a sample of subjects who had previously been observed [16,19]; in the latter, large samples of subjects were observed and interviewed, without the two measures necessarily referring to the same individuals [17,20]. According to such studies, self-reported rates proved higher for females and were always higher than the observational estimates for the same period, as much as twice the observed seat belt usage [16,17,21].

Such studies have shown that the over-reporting factor of self-reported measures is higher in populations with a low observed seat belt use [14,16].

To our knowledge, the extent to which this over-reporting tendency applies specifically to rear seat passengers, who are known to have low seat belt wearing rates, has yet to be investigated.

The aims of this study were:

- to assess the magnitude of over-reporting on self-reported seat belt use among rear seat passengers, when compared with observational estimates;

- to establish whether the over-reporting factor increased after rear seat belt use became compulsory and whether there are any gender-related differences in said factor.

## Methods

The prevalence of seat belt use among rear seat passengers was determined by means of cross-sectional self-reporting and observational surveys conducted in two time periods: in 2003, when rear seat passengers not wearing seat belts were not liable to fines, and in 2005, after failing to wear a seat belt became a legal offence in Italy for all vehicle occupants (June 2003) [22].

The legislation that came into force in June 2003 revised the Italian traffic code and introduced a penalty points system, doubling fines and adding penalty points for unrestrained drivers and punishing all vehicle occupants not wearing a seat belt with fines. The introduction of the new law was accompanied by a state-wide public information campaign, aiming to improve public awareness of the new penalty points system and to increase compliance with the traffic regulations introduced.

The two surveys were conducted in the Veneto Region, a highly industrialized area in the North-East of Italy comprising seven provinces with a population of 4.7 million, and a population density of 258 per km^2^, in 2005.

### Self-report survey

A stratified design for sampling non-institutionalized adults living in households with a telephone was adopted. Telephone numbers in each of the seven Veneto provinces were selected using random digit dialling techniques. Each provincial sample was sized to contain the sampling error to ± 0.05 among a 0.25 expected proportion of seat belt use and a 95% confidence interval (disproportionate sampling). The person to interview was randomly selected within each household contacted.

The safety belt module was part of a slightly modified version of the Behavioral Risk Factor Surveillance System survey, pre-tested on 70 subjects [23].

For the outcome of interest, participants were asked to respond to the following question, "Overall, how often do you wear a seat belt in the rear seats?", using a 6-point scale, i.e. "always", "almost always", "sometimes", "seldom", "never", or "don't know".

Data were collected by trained interviewers using a computer-assisted telephone interviewing (CATI) system. 4002 telephone interviews were completed in May 2003, and 3900 in May 2005 (approximately 1‰ of all adults living in the region) with an overall response rate of 30%, that was similar for the two periods, for the section of the questionnaire referring to seat belt use.

No demographic information could be collected on people refusing to participate to assure anonymity and comply with privacy laws.

The interviews for both periods were conducted mostly in the evenings and at week-ends.

Seat belt use was subsequently analyzed as a binary indicator, i.e. "always used" versus all other categories grouped together, since previous research had demonstrated that results obtained using this approach correlated closely with estimates based on observational data [21].

### Observational survey

A cross-sectional observational study assessed the prevalence of seat belt use among adult rear seat passengers of cars, light trucks and vans, in April 2003 and October 2005.

A multi-stage sample stratification was designed to collect a sample representative of the Region's demography and based on sampling procedures established by the National Highway Traffic Safety Administration (NHTSA) was carried out [24].

The first stage involved selecting municipalities: we chose the 7 provincial capitals plus a random selection of 15 municipalities with probability proportional to province population size. The second stage involved making a random selection of 20 road segments within each of the above 22 sample municipalities. The third stage was a convenience sampling of observation locations within each road segment, to guarantee safety of observers and the reliability of their observations. The same number of observations had to be secured for each road segment selected.

These observation sites included intersections with traffic lights and highway tollbooths, where vehicles could be monitored as they slowed or stopped, enabling accurate data collection.

The sample size was chosen to guarantee a margin of error of ± 1% at a 95% CI (expected proportion of safety belt usage: 10%), with an estimated 3459 observations to be secured for each time period. 4075 rear passengers were observed in April 2003, and 4063 in October 2005.

Emergency vehicles (such as ambulances or police cars), heavy trucks, buses and all vehicles with foreign number plates were excluded.

Observers were selected from health professionals employed by the Local Health Agencies, who took part in an intensive training course and recording practice.

For each eligible vehicle, observers recorded gender and safety belt usage, the survey being conducted during weekdays, from 8 a.m. to 6 p.m. If two or more occupants were seated in the rear, only the rear passenger seated behind the front passenger was observed. Details of the observational study procedures have been extensively reported elsewhere [25].

Gender differences in the prevalence of rear seat belt use were examined using the chi-square test. The over-reporting factor, defined as the ratio of the self-reported to the observational prevalence of rear seat belt use, was calculated by gender before and after rear seat belt use became compulsory.

We used crude data for both types of studies and tested whether the over-reporting factor varied across sex and study year strata by means of the Mantel-Haenszel chi-square for homogeneity, and whether there was a significant interaction term between year and type of study in a robust Poisson regression model.

Ethical approval was not needed for this study.

## Results

The prevalence of seat belt use was recorded for 8138 rear seat passengers in the two observational studies, whereas 7902 interviews were carried out for the two self-report surveys. Table 1 shows the distribution of rear seat passengers observed or self-reportedly wearing seat belts, divided by gender, before and after the introduction of legislation making rear seat belt use compulsory, along with the over-reporting factor calculated for the self-reported versus observational estimates.

**Table 1:** Observational and self-reported data on seat belt use by rear seat passengers by gender and year (2003 and 2005), Veneto Region, Italy

	Observational				Self-reported*				Over-reporting
			
	Sample size	Belted % (95% CI)		P value	Sample size	Belted % (95% CI)		P value	factor
2003									
Male	1653	10.6	(9.1 – 12.1)	0.276	1869	13.5	(12.0 – 15.1)	0.001	1.3
Female	2422	11.7	(10.4 – 13.0)		2021	17.5	(15.8 – 19.1)		1.5

2005									
Male	1853	25.0	(23.0 – 27.0)	0.064	1338	35.8	(33.2 – 38.4)	0.070	1.4
Female	2210	27.6	(25.7 – 29.4)		2410	38.8	(36.9 – 40.7)		1.4

* Percentages calculated without missing data (112/4002 for 2003; 152/3900 for 2005)

According to the observational study, seat belt use rose from 10.6% (95% CI 9.1–12.1) to 25.0% (95% CI 23.0–27.0) among males and from 11.7% (95% CI 10.4–13.0) to 27.6% (95% CI 25.7–29.4) among females in the interval between 2003 and 2005. The self-reported rates were always higher than the observational figures, up to 1.5 times higher in the earlier time period. After the use of rear seat belts become compulsory, their usage was over-reported by 10.8% and 11.2% of males and females, respectively, corresponding to a factor of 1.4 in both cases.

Gender differences were significant (p < 0.05) for the self-reported estimates referring to the first period, but not for the observational findings.

Changes over time in the over-reporting factor were not statistically significant by either the Mantel-Haenszel chi-square for homogeneity or the Poisson regression model (data not shown).

## Discussion

The self-reported rates of belt usage by rear seat passengers overestimated the observational findings both before and after they became enforced with heavy fines. However, the magnitude of the tendency to over-report seat belt use did not increase in the latter period.

Our findings referring to rear seat occupants are comparable in direction and magnitude with those reported on front seat occupants. Previous research focusing on self-reporting telephone surveys and observed seat belt usage revealed an over-reporting factor ranging from 1.2–2.0 in countries without legislation making seat belts compulsory, as opposed to a factor of 1.2–1.4 where such legislation was in place, suggesting that self-reported belt usage estimates should be cut by 10–12% to approximate actual belt use [16,21].

In line with these figures, we found that self-reported estimates were 10–11% higher than observed rates after rear belt use become compulsory.

Several authors found that over-reporting was higher in populations making less use of seat belts [14,16]. We found a higher over-reporting factor for females than for males in 2003, when the rate of seat belt use in Italy was still extremely low.

The law enforcement for promoting rear seat belt use did not increase the factor by which respondents over-reported their use of seat belts, however, and this is consistent with previous findings [16].

A slightly greater tendency to over-report seat belt usage was recorded among females, probably because women are more likely to provide an answer consistent with positive social norms [26].

The present study had several limitations.

First, self-reported estimates might be distorted by respondents' desire to report socially acceptable behaviors, leading to an over-estimation of self-reported seat belt use [14,27]. The aim of the study was to evaluate the extent of such over-reporting, however.

Second, there may have been sampling biases involved in both the methods we used. It may be that the observational study captured people who travelled more often in the rear seats of the vehicles than among the adults answering the telephone survey, who might have a different attitude to the use of rear seat belts; this could have an impact on the reported rate of seat belt use, consequently affecting the estimates of the over-reporting factor. Moreover, the response rate in our self-reported survey was low, and we cannot say whether the people refusing to answer our questions or not at home had the same characteristics as those who took part in the survey.

According to the Italian Personal Data Protection Code, record linkage procedures enabling the identification of basic demographic details of people refusing to be interviewed are now strictly forbidden [28]. The "healthy volunteer effect" that applies to occupational and clinical epidemiology can nonetheless be considered in this setting. It may be that "healthy" people (i.e. people more conscientious about their health) are more compliant in responding to such surveys, directing self-reported estimates towards higher values than those measurable in a random sample of subjects.

Third, self-reported data were obtained using telephone interviews and were thus restricted to telephone owners and to non-institutionalized, civilian adults, who might not be representative of safety belt usage among younger people, institutionalized elderly people, or military personnel.

Fourth, even if the samples used for the two surveys were judged to be representative of the population in our region, our findings may only be referable to this part of Italy and may not be generalizable.

Finally, observational and self-reported measures were obtained at different times of the year. However, previous research has shown that seatbelt use is unaffected by the seasons in this area, so this is unlikely to bias the estimates [29].

The low rate of response for the self-reported survey seems to be the major weakness of this study. The over-reporting factor estimated in our study, in spite of a possible overestimation due to the low response rate (assuming that responders have a healthier behavior than non-responders), is similar, however, to the one reported for front passengers, contributing to our knowledge relating to rear passengers.

## Conclusion

Self-report surveys on belt use by rear seat passengers represents an efficient alternative to costly observational studies and might be useful for evaluating the impact of seat belt laws and road safety measures, tracking changes in actual behavior. Self-reported rates need to be adjusted by an appropriate over-reporting factor, however, in order for them to represent actual seat belt use accurately.

## Competing interests

The authors declare that they have no competing interests.

## Authors' contributions

FZ contributed to the conception and design of the study. FZ and UF took part in data analysis and interpretation. MM contributed to the study design and data analysis. ES, AF and PS critically revised the manuscript. All authors have read and approved the final version of the paper.

## Pre-publication history

The pre-publication history for this paper can be accessed here:


